# Measurement of resilience potential - development of a resilience assessment grid for emergency departments

**DOI:** 10.1371/journal.pone.0239472

**Published:** 2020-09-21

**Authors:** Sheuwen Chuang, Ju-Chi Ou, Erik Hollnagel, Sen-Kuang Hou

**Affiliations:** 1 Graduate Institute of Data Science, Taipei Medical University, Taipei, Taiwan; 2 Health Policy and Care Research Center, Taipei Medical University, Taipei, Taiwan; 3 Department of Emergency Medicine, Shuang-Ho Hospital, Taipei Medical University, Taipei, Taiwan; 4 School of Health and Welfare, Jönköping University, Jönköping, Sweden; 5 Department of Emergency Medicine, Taipei Medical University Hospital, Taipei Medical University, Taipei, Taiwan; Northeastern University, UNITED STATES

## Abstract

**Background:**

Resilience engineering has been advocated as an alternative to the management of safety over the last decade in many domains. However, to facilitate metrics for measuring and helping analyze the resilience potential for emergency departments (EDs) remains a significant challenge. The study aims to redesign the Hollnagel’s resilience assessment grid (RAG) into a custom-made RAG (ED-RAG) to support resilience management in EDs.

**Methods:**

The study approach had three parts: 1) translation of Hollnagel’s RAG into Chinese version, followed by generation of a tailored set of ED-RAG questions adapted to EDs; 2) testing and revising the tailored sets until to achieve satisfactory validity for application; 3) design of a new rating scale and scoring method. The test criteria of the ED-RAG questionnaire adopted the modified three-level scoring criteria proposed by Bloom and Fischer. The study setting of the field test is a private regional hospital.

**Results:**

The fifth version of ED-RAG was acceptable after a field test. It has three sets of open structured questions for the potentials to respond, monitor, and anticipate, and a set of structured questions for the potential to learn. It contained 38 questions corresponding to 32 foci. A new 4-level rating scale along with a novel scaling method can improve the scores conversion validity and communication between team members and across investigations. This final version is set to complete an interview for around 2 hours.

**Conclusions:**

The ED-RAG represents a snapshot of EDs’resilience under specific conditions. It might be performed multiple times by a single hospital to monitor the directions and contents of improvement that can supplement conventional safety management toward resilience. Some considerations are required to be successful when hospitals use it. Future studies to overcome the potential methodological weaknesses of the ED-RAG are needed.

## Introduction

Resilience engineering (RE) has been advocated as an alternative to the management of safety over the last decade in many domains. RE marks a philosophical as well as practical shift towards a proactive systems approach that addresses the need for organizations to cope with the changes in the environment as well as the need for workers to cope with the variations in everyday working conditions [1–3]. Healthcare practitioners are generally forced to adapt their actions to cope with the complexity of daily conditions, especially in emergency departments (EDs). The benefits of RE are conceptually discussed in health care, and resilience applications have been extended to health care in recent years [4–6].

Resilience can be defined as “the intrinsic ability of a health care system (a clinic, ward, a hospital, a country) to adjust its functioning prior to, during, or following events (changes, disturbances, and opportunities), and thereby sustain required operations under both expected and unexpected conditions” [5, 7]. This definition requires possessing the potentials to respond, monitor, anticipate, and learn. The potential to respond looks at what is needed for an organization or a system to respond timely and effectively to what happens. The potential to monitor considers how well the organization can detect changes to work conditions, and the indicators used to keep track of what happens in a system and its environment as well as whether they are meaningful, effective, and properly maintained. The potential to learn looks at what the learning bases and targets are, when the learning takes place, and how the effects of learning are verified and maintained. And the potential to anticipate addresses an organization’s ability to look ahead and to consider future events, conditions, threats and opportunities [7, 8].

The four potentials of resilience (responding, monitoring, anticipating, and learning) have contributed to creating a wide consensus on the resilience structure [3]. The four potentials for resilient performance can be used as the basis for four coherent sets of markers or proxy measurements from which an assessment can be made. To facilitate a better understanding of what makes resilient performance possible, the resilience assessment grid (RAG) that consists of four sets of generic questions was developed by Hollnagel as a basis for collecting these proxy measurements. The four sets of questions were used to construct a resilience profile for each of the four resilience potentials [7, 8]. The RAG demonstrates an idea and guideline for measuring resilience by considering the potentials for improving resilience. It requires the analyst to adjust its structure to the organization or system being studied for wider applications, such as, the RAG questions were tailored to create questions for exploring the potentials in construction processes [9], measuring the resilience of anaesthesiologists [10], and investigating organizational resilience in the water sector of England and Wales [11].

Emergency Departments (EDs) are dynamic, open, high-risk systems that function under considerable uncertainty and economic pressure [12, 13]. Their ability to perform in a resilient manner can, therefore, be critical. EDs have been considered particularly well-suited to investigating resilience in action [14]. A growing number of articles have discussed resilient health care in emergency departments, and recognize that to operate effectively and create value, EDs must be flexible, having the ability to rapidly adapt to the highly variable needs of patient [15, 16]. A fundamental question in ED’s resilience performance is how to assess resilience and implement measures that enhance the EDs’ potentials to sustain required operations under both expected and unexpected conditions. However, the identification of metrics and standards for measuring organizational resilience in health care remains challenging.

The RAG is a relatively new questionnaire-based tool to support resilience management, which has currently not been widely used in healthcare. A limited number of studies were found in literature, e.g., ED resilience study by Hunte and Marsden [17], studying the resilience of anaesthesiologists [10], and creating an interview script to interview physician and nurse-providers about resilience in health care [18]. We view the absence of easy-to-use operational measures as one of the key barriers to the improvement of resilience in EDs. The study drew on the four potentials and their corresponding sets of generic questions of the RAG to further redesign an appropriate RAG specifically aiming at the assessment of EDs’ resilience potentials, hence named ED-RAG. Thus, the ED-RAG can help clinicians and managers to better understand the EDs’ resilience potentials to manage ED operations toward resilience under the context of conventional safety management. This paper presents the result of ED-RAG and the implication of its utilization.

## Methods

The study approach had three parts: 1) comprehensive understanding and translation of Hollnagel’s RAG into Chinese version, followed by generation of a tailored set of ED-RAG questions adapted to emergency departments; 2) testing and revising the tailored sets until to achieve satisfactory validity for application; 3) redesign of RAG scoring method into a new rating scale and scoring scheme for the ED-RAG.

### Generic resilience assessment grid

The RAG is an open structured questionnaire organized into four sets of generic questions, one for each potential. The general version comprises 39 foci and 64 questions (including subquestions in each question) addressing a variety of foci related to each of the four potentials [7, 8]. Foci represent multiple focuses. Each focus is individual dimension contributing to a resilience potential, such as the focus “event list” in the potential to respond. Each focus may have one or more questions to diagnosis the related potential. Responses to each question can be short or long sentences and are translated into the following five-level Likert-type ordinal scale answers: namely excellent (5), satisfactory (4), acceptable (3), unacceptable (2), and deficient (1). At one end of the scale, “excellent” means the system exceeds and meets the criteria. At the other end, “deficient” means that a system has insufficient capability. The answers then can be combined to provide a profile for each potential as well as an overall profile of the system’s potentials for resilient performance.

Regarding measuring resilience potentials in EDs, a tailored set of RAG questions should be adapted based on relevance and importance to emergency departments. Each focus and its associated questions need to be examined by the subject experts or cross-checked using the relevant ED documents, e.g., emergency response plans (ERPs), quality and safety management policies and procedures. For example, the focus “event list” and “response list”in the potential to respond can be found in ERPs, where “event list” indicates the events might occur in EDs, e.g., power outage, mass casualty incident, the “response list”shows actions taken to each corresponding event. The RAG 39 foci were described in Hollnagel’s publications [7, 8], while the description of the selected foci of ED-RAG can be found in S1 Appendix.

### Test criteria of the ED-RAG questionnaire

It is important to gain a strong consensus among the subject experts in the process of defining the final set of questions, since this will clearly affect the definition of the resilience profile [19]. The inter-rater reliability (IRR) that assesses how well multiple raters provide similar ratings was evaluated by Fleiss’ Kappa [20]. The value above 0.8 indicates almost perfect agreement, and the value below 0.2 indicates weak or slight agreement. The various versions of the ED-RAG questionnaire were tested according to the questionnaire design criteria of purpose, directness, and utility proposed by Bloom and Fischer [21].

Each question was evaluated in the content of“Relevant to ED environment,” “Importance to ED,” and “Clarity” (Table 1) based on the modified scoring criteria of Windle et al. [22]. A score of 2 points was awarded if the criterion was met completely; 1 point if the criterion was partially met; and 0 points if there was no evidence or if the criterion was not met. The average Windle’ content validity score of questionnaires was calculated for summarizing the content validity across questions. The worst and perfect average Windle’s content validity scores were 0 and 2.

**Table 1:** Scoring criteria for the content validity (modified from Windle et al. [22]).

Score	Relevant to ED environment	Importance to ED	Clarity
**2**	The extent to which the focus of interest is comprehensively relevant to the ED working environment and specified by the questions within each focus.	Target objects in each question were involved in building of specified potential	A clear description of measurement aim
**1**		A doubtful description	Lacking a clear description
**0**		No involvement	No information found

### Design and test of ED-RAG

**Fig 1:**
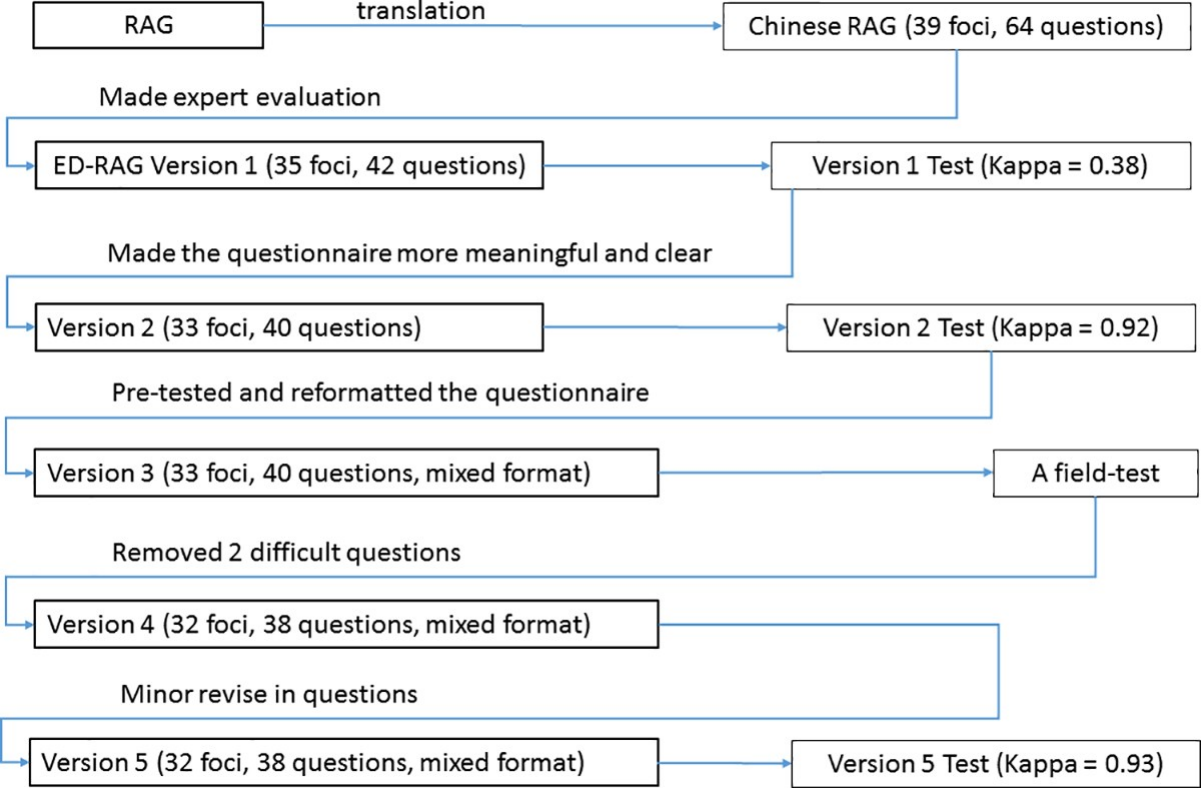
Design and test flow of the ED-RAG questionnaire.

The design and test of the ED-RAG questionnaire is briefly described in five versions (Fig 1) as follows.

#### Version 1

After thorough discussion with the RAG’s developer, the RAG questionnaire was comprehensively understood and translated into a Chinese version. The Chinese version was used as the base of working on the development of version 1 by a cross-disciplinary research team. The team consisted of four individuals: an ED director; a health care faculty; a post-doctoral researcher working in ED, and a research assistant. Development of the Version 1 started with comparing the RAG Chinese questionnaire with the corresponding ED relevant policies and procedures of three hospitals, followed by selecting proper questions and rewriting questions for each potential set. This step produced 42 specific questions in four sets of an ED-RAG questionnaire. To achieve the consensus of an acceptable questionnaire, the version was assessed by three ED physicians via the modified criteria of Windle et.al [22]. Findings from the average Windle’s validity score at 1.44 and Kappa statistics at 0.38 show that this version with only fair content validity and fair IRR agreement. Therefore, the second version of ED-RAG was generated according to the suggestions.

#### Version 2

The development of Version 2 made the questionnaire more meaningful and clear for ED working environment according to the test result and experts’ comments in the version 1. Forty out of 42 questions were determined to be relevant, important, and feasible. For example, there are two questions about the focus "time horizon" of the potential to anticipate. The one "Is the time horizon different for ED business and safety?" was deleted in version 1. The last one in this focus, "How far ahead does the ED look ahead in daily work?" was removed in version 2. Reviewers' comments included "not clear, ED always looks ahead," "by situations, responses could be many," "difficult to answer." Therefore, the focus "time horizon" was taken out of the ED-RAG questionnaire. This version was tested by the three physicians (Kappa = 0.92, average Windle’s content validity score = 1.9). Version 2 then was used to conduct a pretest. This test is to demonstrate the version 2’s understandability and determine the length of interviews, as well as to improve questioning skills. The interview was undertaken by the lead researcher to interview a post-doctoral researcher who was a senior interviewer in medical field.

#### Version 3

Since Version 2 required more than 2 hours for an interview, a third version was developed by mainly tuning the questions’ sequence to the interviewee’s understanding, and transformation of questions format to satisfy the pre-defined maximum 2 hours interview time. The questions of potential to learn were determined to change from open-ended to structured questions based on the findings that relatively more specific answers to the questions of potential to learn and less variation in learning processes than the corresponding answers to the other three potentials were identified in this test.

The reformulation was conducted by analyzing the interviewee’s answers and comments, and searching for possible additional answers corresponding to each question in the potential through examining and comparing the three hospitals’ training policies, procedures and activates documents relevant to quality and patient safety management. This produced a set of answers of short phrases corresponding to each question. For example, to answer the question “How are the events selected for learning purposes? The answers included: Individual departments select independently; Hospital accreditation standards; Events that occur most frequently or are the most serious; Events that occur most frequently and are the most serious. The answers then were listed as a set of selection items, respondents pick one or multiple items when they answer the question. The version 3 thus was composed of 3 open ended questions sets for the potential to respond, monitor, anticipate and one structured set of questions with multiple choices for the potential to learn. This version was approved by the Taipei Medical University—Joint Institutional Review Board.

#### Version 4

To obtain the knowledge from the field’s response to the questionnaire, Version 3 was tested by an interview with a focus group from the ED of a participating hospital. This was a private hospital with approximate 3.500 ED patient visits annually. The focus group included the ED director, the vice director who was responsible for the ED’s administration, a physician, and a head nurse. This field test was to identify what questions were difficult to answer by ED practitioners, as well as to test the viability of the survey as a whole. The test removed two questions of the focus delay in the potential to learn that were challenging to answer. The question “what is the delay in reporting and learning?” raised various aspects to the definition of delay in the focus group. Therefore, the question “Regarding the delay, how are the outcomes communicated internally and externally?” was also confused and hardly answered.

#### Version 5

Version 5 was revised based on the responses of the focus group. This step finalized 32 foci and 38 questions, and slightly adjusted the excerpts in the potential to learn. For example, to answer the question “How are the events selected for learning purposes?”, an original excerpt is “Individual departments select independently,” the revision is “Individual departments select independently (no rule)”; to answer the question “Are there any formal procedures for data collection, analysis, and learning?” an original excerpt is “Analysis with less training,” the revised answer is “Analysis only without training.” The purpose of revision is to provide clear meaning to each option. Respondents answer the question by choosing one or multiple excerpts out of the four options. The final version was evaluated by three professionals, two ED physicians, and one health care faculty with an almost consistent agreement (Kappa = 0.93, average Windle’s content validity score = 1.98).

### Development of a new rating scale and a new scoring method

#### Four-point scale

During the questionnaire design, no consensus was reached among the research team members on the definitions of the RAG five classes: excellent (5), satisfactory (4), acceptable (3), unacceptable (2), and deficient (1). For example, the RAG named the highest level is “excellent”, this word represents extremely good; however, few raters knew what the excellent state was to individual questions measuring the resilience potentials under the stage of preliminary development of ED resilience. Besides, the difficulties of classifying unacceptable and deficient performance were identified during the field test. Moreover, insufficient evidences (answers) collected at the initial stage could hardly be classified into five levels. To reduce the ambiguity caused by the five classifications' drawbacks, the study adopted a four-point (1 to 4) scale. There were no pre-defined specific names, e.g., excellent or unacceptable for the four levels. The answers to each question were objectively compared, and a higher score indicated relatively better performance in the comparison.

The point (1 to 4) is not shown in the questionnaire but determined after a survey. Respondents’ answers demonstrate the situation of ED fitting to the questions of the four potentials. The answers collected from the structured questions and the responses to the open-end questions were coded into 1–4 accordingly afterward. An Excel file was designed with a specific rating algorithm for each question in the potential to learn, and scoring and drawing methods for generating the radar and star charts. It was used to support the ED-RAG rating, scoring and plotting functions, and storing the collected answers at the time of measurement as a base for continuously refining the measurement of ED resilience potentials.

After responses were collected, two researchers engaged in the coding and synthesizing processes for individual answers corresponding to each question. The transcripts of the answers to the three sets of open-end questions were analyzed using thematic analysis. The answer list of selected option(s) for the structured questions was analyzed by comparing each set of answer(s) to its corresponding question's predefined rating algorithm. Each rating algorithm was synthesized and agreed upon by the participated researchers, and the content of the collected answers determined its score (1–4). Examples of the four-point levels are described in Table 3. The rating algorithms to the questions of potential to learn may be changed if additional data are engaged.

For the thematic analysis, the researchers discussed the steps of analysis approach before engaging in the steps independently, followed by reading through entire transcriptions and identified codes from significant phrases and sentences in individual answers. After completing the synthesis processes, according to the MECE (mutually exclusive and collectively exhaustive) principle [23], the researchers categorized these codes into four labels for individual questions. The two researchers met again to compare their emerged labels and contents, and refined and named the labels as a four scale level (1, 2, 3, 4) for rating the answers to individual questions. After the agreement for the definition of each scale level derived from their coding and categories of contents was achieved, the two researchers then assigned a specific level (from 1 to 4) to each question for the following score calculation purposes. However, the classification did not always provide exact thresholds to determine when the performance changes from one potential resilience level to another. With the presence of a third researcher, any disagreements were discussed openly to reach a consensus on the scale code.

#### Scoring method

The RAG does not present a transparent conversion formula that transforms the multiple focus scores of individual potentials in the corresponding radar chart into an aggregated score of individual potentials in the star chart. The study developed a new scoring method to establish the connection between the two measures and link to an overall aggregation score of ED system resilience. The calculation of individual potential score and the overall score of ED system resilience potential relies on computing algorithms rather than human judgment. The new scoring method intends to reduce the validity issue’s effect on the measurement.

This new method includes calculation of average score per focus (focus score), score of potential, and aggregation score—overall score of ED system resilience. A focus may contain one or more questions, and the average score of a set of questions in each focus (focus score) represents the performance of the focus. Each resilience potential contains several foci. The score of potential is the sum of multiple focus scores—the size of the covered area in a radar chart, rather than a mean value of foci for individual potentials. Because the four-point scale is still treated as an ordinal scale, to calculate the focus score may create an argument that ordinal data cannot yield mean values. There are only 19% (6/32) foci have two questions in the questionnaire, other focus has just one question (S1 Appendix). Therefore, the study adopted calculation of means only for the average score per focus in the 6 foci based on the understanding that every question to a focus was considered to be of the same importance. The limited numbers of foci using the average score per focus provide relatively easy understanding to users and less negative effects on scale validity.

**Fig 2:**
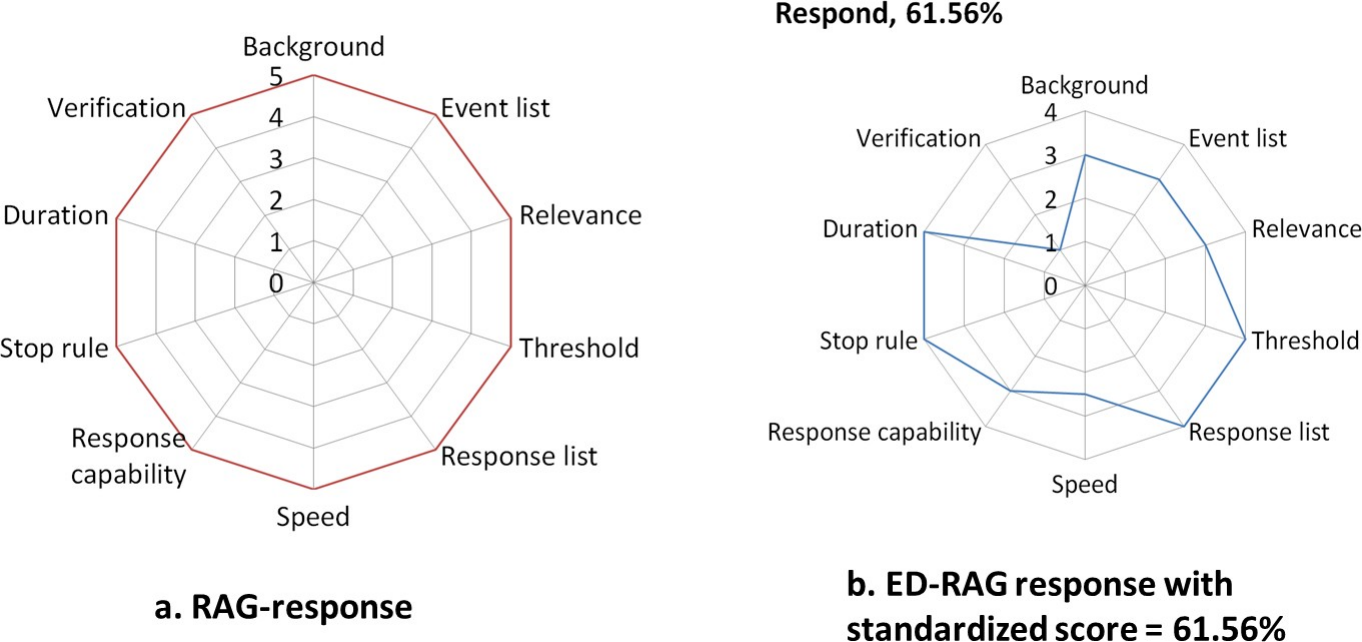
Five-level scale of RAG and four-level scale of ED-RAG in the potential to respond.

In the ED-EAG, a series of focus scores relating to a potential was calculated separately and then plotted them in fixed positions on a radar chart. It was seen as desirable to represent a potential, including numbers of foci, by a single number. This was obtained by calculating the covered area of the radar graph for each potential (Fig 2). The area of a polygon was determined as the sum of the areas of all triangles in a radar chart; the area of each triangle was determined by multiplying the lengths of two adjacent sides by the sine of included angle and then divide by 2. The size of the calculated area (score of potential) was used to represent literally the capability of each potential. A standardization process for the calculated area of each potential was adopted to convert the area's size into a percentage number to eliminate the comparison biases between different radar charts and to improve understanding of the capability scale. The standardized potential score is defined as the percentage of the total area of each potential in its radar chart. Then this standardized potential score was plotted in a fixed potion on the star chart of overall system resilience.

**Fig 3:**
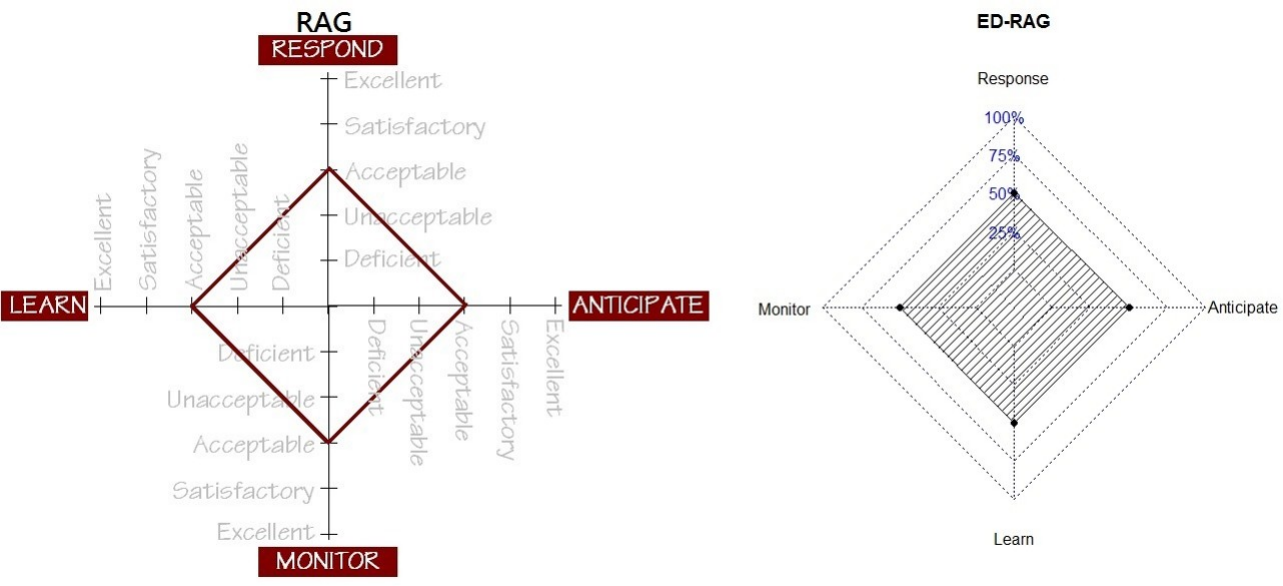
RAG star diagram with verbalized ratings vs ED-RAG chart with numeric ratings.

Besides, a single number called an aggregation score—the overall score of ED resilience potential was proposed to represent the system’s potential for an ED resilience. It is generated by calculating the total of four triangle areas from the resilience star chart (Fig 3). This aggregation score also can be standardized and ranges from 0% to 100% (0 to1), where a higher percentage indicates better system potentials.

## Results

### The ED-RAG foci and questions

The latest version of questionnaire (Version 5) contained 38 questions corresponding to 32 foci in four potentials, compared to the 39 foci in the RAG. The version 5 questionnaire is attached as S1 Appendix. Table 2 shows a comparison between the RAG and the ED-RAG in terms of the number of foci and questions corresponding to each potential. In the ED-RAG, eight foci were removed from the RAG while one focus, namely indicator characteristic, was amended. The eight removed foci were “indicator type”, “measurement type”, “stability”, and “delay” for the potential to monitor; “delay” for the potential to learn; “time horizon”, “acceptability of risks” and “aetiology” for the potential to anticipate. These foci were removed either because they were impracticable or hard to apply properly or difficult to answer under EDs’ setting.

**Table 2:** Comparison of foci between RAG and ED-RAG (Y or N /number of questions).

Potential	Focus	RAG	ED-RAG	Potential	Focus	RAG	ED-RAG
Respond	Background	Y/1	Y/1	Learn	Selection criteria	Y/3	Y/1
	Event list	Y/1	Y/1		Learning basis	Y/1	Y/2
	Relevance	Y/3	Y/1		Formalization	Y/1	Y/1
	Threshold	Y/2	Y/2		Learning style	Y/1	Y/1
	Response list	Y/2	Y/1		Resources	Y/3	Y/2
	Speed	Y/1	Y/2		Implementation	Y/2	Y/1
	Response capability	Y/2	Y/2		Training	Y/1	Y/1
	Stop rule	Y/1	Y/1		Classification	Y/2	Y/1
	Duration	Y/1	Y/1		Learning target	Y/1	Y/1
	Verification	Y/2	Y/1		Delay	Y/2	N
Subtotal	foci/questions	10/16	10/13	Subtotal	foci/questions	10 /17	9/11
Potential	Focus	RAG	ED-RAG	Potential	Focus	RAG	ED-RAG
Monitor	Indicator list	Y/1	Y/1	Anticipate	Frequency	Y/1	Y/1
	Indicator characteristic*	Y/0	Y/1		Model	Y/2	Y/1
	Relevance	N/4	Y/1		Communication	Y/1	Y/1
	Measurement frequency	Y/1	Y/1		Expertise	Y/1	Y/2
	Organizational support	Y/2	Y/1		Strategy	Y/1	Y/1
	Analysis / interpretation	Y/3	Y/1		Culture	Y/1	Y/1
	Validity	Y/2	Y/1		Acceptability of risks	Y/2	N
	Stability	Y/1	N		Time horizon	Y/1	N
	Measurement type	Y/2	N				
	Delay	Y/2	N				
Subtotal	foci/questions	10/20	7/7	Subtotal	foci/questions	9/11	6/7

Y: Included, N: Excluded.

*: Emerged focus from modifying the questions in the focus Indicator type, Measurement type, and Delay.

### ED-RAG rating scale and scoring method

The ED-RAG uses a scale with four levels called 1, 2, 3, 4. Level 4 represents the expected relatively best performance to the question. Level 3, 2, 1 may have different interpretations according to the nature of the performance addressed by each question. Table 3 indicates three examples. The first one is the rated four levels of answers to the open-end question “Have you prepared the necessary resources and manpower for routinely verifying or maintaining your emergency response plan and procedures?” The other two show the rating algorithms of two structured questions. Each level 4 is the relatively best performance among the answers to the corresponding questions. But it does not mean “excellent.”

**Table 3:** Rating scale examples—an open-end question and two structured questions.

Four-point scale	Answer(s)
Open-end question: Have you prepared the necessary resources and manpower for routinely verifying or maintaining your emergency response plan and procedures?	
4	Periodically maintain and verify the configured resources for expected emergency cases
3	Consult experts for verifying the needed resources once in a while
2	Reconfigure the needed emergency resources after something happened
1	Never verify or change the planned resources for emergency cases
Structured question: How much attention does the hospital pay to employees’ suggestion(s)	
4	Respond to all suggestions and require a clearer explanation from the employees
	Discuss as soon as possible for all suggestions
3	Respond to the feasible suggestion(s)
	Discuss as soon as possible for all suggestions
2	Respond to the feasible suggestion(s)
1	No response to the feasible suggestion(s)
Structured question:	How do you collect events for learning?
4	Based on department needs, based on hospital needs, based on hospital accreditation needs
3	Any two of the above three options.
2	Any one of the above three options.
1	None

After a scale level was determined for each question, each focus score was calculated. For example, the focus “learning basis” in the potential to learn has two questions. The test hospital obtained a score level 4 (Respond to all suggestions and require a clearer explanation from the employee, and Discuss as soon as possible for all suggestions) to the question “How much attention does the hospital pay to employees’ suggestion(s)?”, and another level 4 to the question “Does the system try to learn from success as well as from failures?”. Therefore, the focus score is (4 + 4)/2 = 4. Next, each focus with a corresponding focus score was posted on the radar chart of individual potentials accordingly. The scoring method is illustrated for the potential to respond in Fig 2, which shows the ED-RAG four-level scale. The area calculated for this potential represents its standardized score of potential, which is 61.56%.

### Aggregated resilience of ED-RAG

Whereas the RAG uses verbalized abilities in five-level scale, the ED-RAG transforms each score of potential calculated from the occupied area in each radar graph into a standardized score (0 ~ 100%), e.g., 61.56% for the potential to respond, 33.93% for the potential to monitor, etc., based on the method described in the method section. A system’s potential for overall resilience: overall score of ED system resilience, can, therefore, be represented by a star chart with the score of percentages (%) in four directions. The comparison of the aggregated score of system resilience between RAG and ED-RAG is shown in Fig 3. The ED-RAG therefore links the focus score, score of potential to the overall score of system resilience.

### Use of the ED-RAG and its interpretation

**Fig 4:**
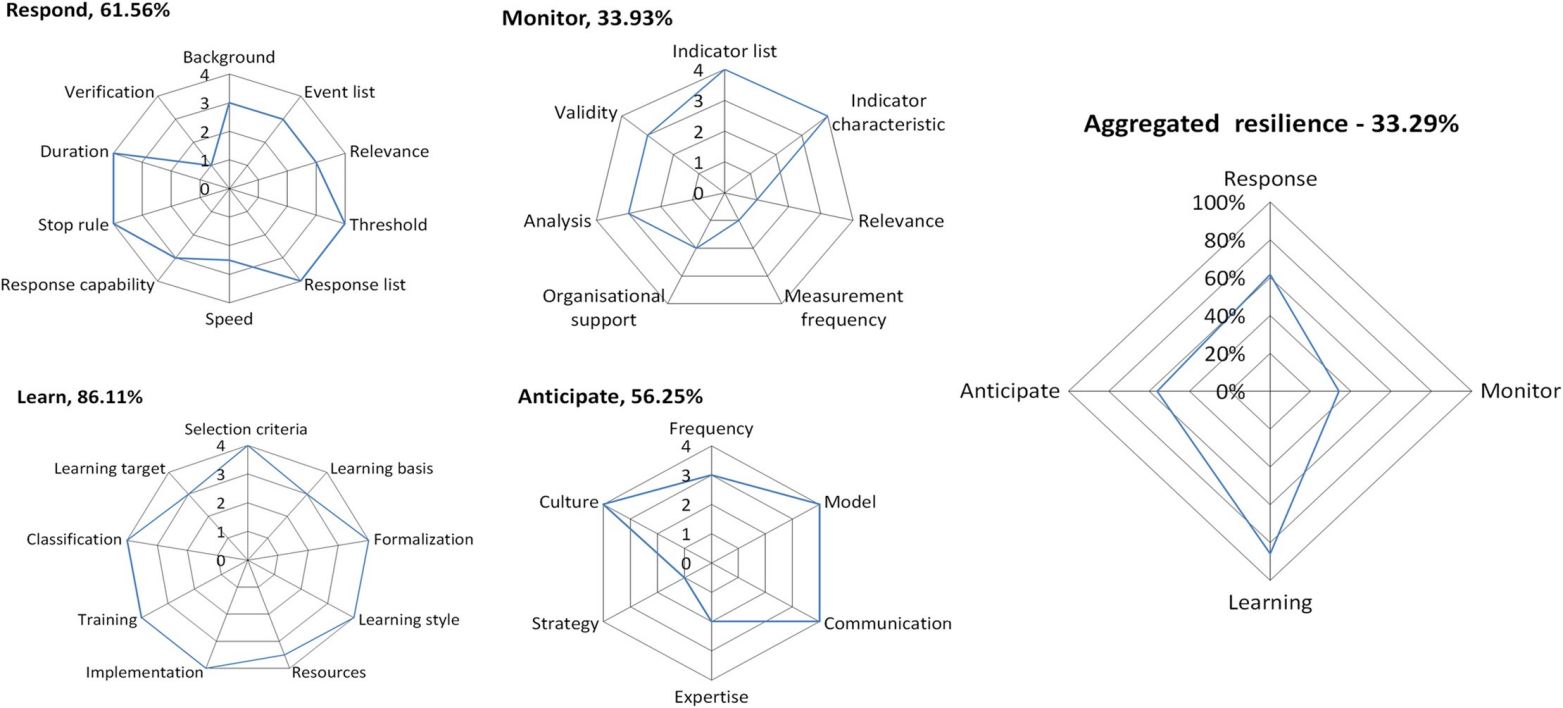
The field test case: Four potentials’ radar charts and overall resilience score chart.

The ED-RAG (version 4) was tested in one participant hospital. Its corresponding radar charts and star chart are shown in Fig 4. Based on the proxy measurement of resilience of the ED using the ED-RAG, the hospital had better potential to learn (score of potential = 86.11%), followed by response (6156%), anticipate (56.25%) and monitor (33.93%), at the time of measurement. Their radar charts provide visualized results that indicate clear directions with relatively low focus score for making an improvement plan toward resilience in ED. The detailed scores are shown in Table 4.

**Table 4:** Detailed scores of the test case.

Potential	Focus	Number of question	score
Response	Background	1	3
	Event list	1	3
	Relevance	1	3
	Threshold	2	4
	Response list	1	4
	Speed	2	2.5
	Response capability	2	3
	Stop rule	1	4
	Duration	1	4
	Verification	1	1
Score of potential to respond			61.56%
Potential	Focus		
Monitor	Indicator list	1	4
	Indicator characteristics	1	4
	Relevance	1	1
	Measurement frequency	1	1
	Organizational support	1	2
	Analysis	1	3
	Validity	1	3
Score of potential to monitor			33.93%
Potential	Focus		
Learn	Selection criteria	1	4
	Learning basis	2	3
	Formalization	1	4
	Learning style	1	4
	Resources	2	3.5
	Implementation	1	4
	Training	1	4
	Classification	1	4
	Learning target	1	3
Score of potential to learn			86.11%
Potential	Focus		
Anticipate	Frequency	1	3
	Model	1	4
	Communication	1	4
	Expertise	2	2
	Strategy	1	1
	Culture	1	4
Score of potential to anticipate			56.25%
**ED system resilience**			33.29%

The score of potential to respond to this hospital was 61.56% with score 4 at the following foci: threshold, response list, stop rule, and duration, but the verification was low. The score of potential to monitor was 33.93% with two foci at score 3 (analysis and validity) and two foci at score 1 (relevance, measurement frequency). The score for the potential to learn was 86.11%, with most foci at score 4, except the learning target at score 3. The score for the potential to anticipate was 56.25%, with three focus at score 4 (model, communication, and culture), and the focus strategy performed worst at score 1. The hospital can check the corresponding foci in each potential to identify which focus needs to be improved at the time of measurement. The four potentials to resilience identified by the ED- RAG do not mean the ED resilience performance, but what the ED system did in the four potentials to be resilient.

## Discussion

Following the need to measure resilience for assisting the implementation of the four potentials in EDs, the paper demonstrates how the generic RAG was redesigned into the ED-RAG. It also describes the work to overcome the challenges troubled in the stage of questionnaire design, as well as the scene of application: interview, data analysis, and communication, which included the selection of adequate and feasible questions adapting to ED system, use of the language/words that ED staff can understand, reduction of interview time by redesigning part of the questionnaire into structured questions while maintaining its validity, use of the four-level rating scale to objectively classify the insufficient collected data, and development of scoring methods to enhance the efficiency of communication. Furthermore, this study's findings have highlighted that applications of the RAG or ED-RAG need to consider the following themes to be successful.

### Use of rating scale

The ED-RAG questionnaire is a mix of three sets of open-structured questions and a set of structured questions. It was designed to save the interview time; however, this creates a challenge as the data have to be analyzed differently. The study used the four-point scale (1 to 4) to present the resilience potential of each question. Thematic analysis is suggested to analyze the answers to open-ended questions, and each question will be assigned a specific point (1 to 4) according to the agreement among researchers or investigation team members, while during the interviews, the interviewees are asked to agree on one or more options (item 1–4) in each question for the potential to learn. The study developed an Excel file, which is a resolution to the challenges of mixed data analysis. The Excel file outputs a proper level via a specific rating algorithm to each question in the potential to learn after the answers are inputted. It also stores the thematic phrases and their corresponding code assigned by the analysts for the open-end questions. The rating algorithms should consider being revised if more evidence shows the current 4-level classification is no longer fit the collected data.

The four-point level is an ordinal scale representing an objective classification of activities by a relatively superior sequence rather than a fixed expression, i.e., excellent, satisfactory. At the current stage, utilizing the RAG has not been widely accepted by organizations. There is no agreed or standard definition of the excellent, satisfactory, acceptable, unacceptable, and deficient performance in resilience. These definitions need to be developed and recognized for promoting RAG applications. Clustering the limited number of answers into four levels through repeatedly utilizing the ED-RAG could help practitioners understand what activities to resilience potentials are relatively better than others in EDs, and may gradually extend the knowledge of resilience potentials in five levels, and ultimately develop a consensus of 5-level standard definitions for applications in practice.

Additionally, use of the four-point scale to rate the answers for each question, although the rating process for the structured questions in the potential to learn can automatically generate a proper level code for each question in the Excel file, the time burden associated with the thematic analysis still requires large efforts to assess the open-end questions in other three potentials. Many hospitals would not have qualitative researchers routinely employed who could be used for this purpose. Moreover, the inherent weaknesses in the scale validity of RAG (5 levels) and ED-RAG (4 levels) is another concern while applying the tools. These concerns might raise two perspectives: determination of scale level between investigation team members and interviewees, and mixed-method analysis for generation of scale data. It is essential to know that RAG or ED-RAG applications rely on truthful answers and reliable ratings. The selection of investigation team members and interviewees should be careful.

### New scoring method to improve resilience measurement

The study proposes a novel scoring method to avoid the issue of abusing the ordinal data [24], and to establish a transparent connection approach of linking focus score, potential score and aggregation score of system resilience. This method is a proxy measurement that facilitates a better understanding of the relationships between what the system does in each focus perspective and the presentation of individual resilience potentials and the overall system resilience potential.

The method to calculate the score of potential is to sum the areas of the triangles in the radar chart. Since the original purpose of a radar chart is not to express the areas, but rather the length of the segments (focus), users adopt this method to pay specific attention to the position of each focus on individual radar charts. Because different order of the foci in a radar chart will create different areas, thus a different area size and score of potential, consequently different scores of aggregated system resilience potential. Once users decide to plot each focus on a corresponding radar chart, they should fix the position or order of each focus on the radar chart when a hospital uses it repeatedly for continuous improvement of resilience in its ED.

The ED-RAG can be repeatedly applied by a single hospital for continuous improvement of system resilience in its ED. Although not ideal, it might also be considered as a way to compare resilient performance across hospitals. Users should know that individual potential may have no difference in scores between multiple investigations. However, the score of each focus might be different. In other words, the directions of improvement might be changed. An essential concept of RAG or ED-RAG is to identify the directions and contents for improvement in business processes toward system resilience.

Further, the RAG is intended as a way to get a profile of the four potentials, and to compare these profiles overt time, to follow how an organization develops. The intention was never to use it as a rating tool, neither for a single unit nor to compare multiple units. Although the ED-RAG proposes the scoring method and a single standardized measure for each potential and all potentials together, a potential score or an overall score of ED system resilience does not tell us where the problems/opportunities are, not what to do about them. Chief executive officers may like it because it makes the world look simple, but it is a dangerous illusion. The ED-RAG developed a “code” (the area of a polygon in a radar chart or the score of percentages (%)) rather than “value” in the rating method. The code is used as a symbol to improve the efficiency of communication between team members and across investigations while using the ED-RAG.

### Principles of selecting interviewees or participants

The applications of RAG had been found in an offshore oil and gas company, the air traffic management system, rail traffic management and health care [3]. Several techniques were used to collect RAG data, such as informal phone or face-to-face interviews, focus group discussions, or surveys via e-mail or a website. Literature indicated that the use of open-ended structured questionnaires inherently has a questionable quality of survey data, which could be generated by interviewers or interviewees [25, 26].

The application of RAG or ED-RAG should consider their design nature. The RAG or ED-RAG contains specific detailed questions that help to reveal the preparation states of a system toward resilience [7, 27]. Therefore, the interviewees need to know about the hospital’s activities to provide accurate data. To control the data biases when a hospital wants to repeat ED-RAG applications, the use of consistent interviewer(s) or facilitator(s), as well as keeping interviewees’ consistency in working background every time, is critical. Besides, it is necessary to identify a pool of people working in the system who could answer the questionnaire correctly before implementation.

The principals of selecting interviewees may consider experienced staff in clinical work and administration in EDs. For example, ED director, staff responsible for the ED’s administration, and experienced physician and nurse. However, the number of interviewees is not a critical issue. If the ED participants can spend more time in discussion while conducting the interview or focus group, valuable information could be learned to increase the quality of the interview or credibility of the data.

### Essence of RAG and ED-RAG

Resilience engineering does make clear that a system must harbor the four potentials to be able to perform in a resilient manner, rather depending on conventional safety management that is used to control injury rates/fatality rates plateauing in recent times. Therefore, it is useful to consider the extent to which each of the four potentials for resilient performance is present in or supported by the system to be capable of performing proactive safety management [8, 28]. The ED-RAG demonstrates a novel idea of a scoring method on the basis of RAG. Although it adopts a 4-level instead of a 5-level rating scale, the essence of RAG and ED-RAG focuses on process measures rather than product measures and shows how well the system does on each of the four potentials [22].

The applications of RAG and ED-RAG measure a system’s potentials for resilient performance in a process-based survey. The aims of applying RAG or ED-RAG are first to determine where the system is; then to spot where the system should be, finally to understand how the system may reach a target status [3]. Repeated RAG or ED-RAG profiles can be compared to look for differences, which can be used as the basis for managing the system and following the consequences of planned interventions. The RAG and ED-RAG values are to point out the strengths and weaknesses of the individual resilience potentials and to identify the direction of improvement toward resilient health care. This emerging concept of using ED-RAG to increase resilient performance in EDs may stimulate the evolution of safety management for safer and sustainable operations in health care organizations.

### Study limitation

The development of ED-RAG has served to extend the understanding of resilience abilities in a specific field i.e. EDs, based on the foundations of RAG. However, some issues remain about its current status.

1. The rating scale validity issue is the weakness in the current version. It has the inherent possible biases as being a qualitative survey. The study collected answers from the field test hospital and some relevant documents to generate the rating contents for each question. The method is reasonable in the short term, however, the limited data sources might narrow the classification of rating scale in the long run. More interviews of ED groups from hospitals with different characteristics to enrich the contents for rating scales are needed. In other words, this version as a base of measuring ED resilience and could be improved by further learning from multiple surveys.
2. Regarding the scoring methods, the study currently fixed the plotting positions (order) of the foci and the four potentials on radar charts and a star chart respectively to ensure reproducibility. However, the order itself affects the value of the area obtained; thus, the score of potential and system potential would be impacted. The interactions between foci need to be further studied to prepare for future revisions of ED-RAG.
3. Version 5 of the ED-RAG questionnaire is still time-consuming (around two hours) while surveying with the busy ED staff. Further refinements of the questionnaire, e.g., more open-ended questions transforming into structured questions or determination of critical questions to build the resilience potentials' measures, may be necessary for the wide applications.

## Conclusions

The attention to resilience in EDs has increased markedly over the last decade. However, the identification of metrics and standards for measuring resilience in EDs remains a significant challenge [5]. This study proposes a complete procedure of redesigning the general-purpose RAG into a specific instrument named ED-RAG for ED’s applications. This procedure defined a proper set of questions that would be reliable enough to describe the ED system and developed resolutions dealing with the current drawbacks of RAG and the common weaknesses of RAG and ED-RAG.

The RAG’s distinctive drawbacks to the initial stage of promoting resilience potentials include unsatisfied five-level classification in rating the insufficient collected answers, no agreed standards defining the characteristics of the five levels, no transparent conversion formula presenting the relationships between what the system does in each focus and the individual resilience potentials and the overall system resilience. Besides, RAG and ED-RAG's common weaknesses were identified, including scale validly, long interview time, and time burden associated with the qualitative analysis for hospitals.

Use of the finalized ED-RAG version 5 in the mixed structured questionnaire with a 4-level rating scale and the novel scoring methods and plotting approach initiated an opportunity to overcome the current challenges of measuring resilience in EDs. However, it is still useful to remember that RAG or ED-RAG does not offer an absolute rating of systems abilities, but a relative assessment support tool to compare different potential profiles. The ED-RAG application represents a snapshot of EDs’ resilience potential under specific conditions, should be performed multiple times to follow and monitor the directions and contents for improvement in ED processes toward resilience. Future studies to solve the current methodological weaknesses of the ED-RAG or RAG are needed.

## Supporting information

S1 Appendix ED-RAG questionnaire. (DOCX)
