# Supplementary material for: Measurement of resilience potential - development of a resilience assessment grid for emergency departments
Source: PLoS One. 2020 Sep 21;15(9):e0239472. doi: 10.1371/journal.pone.0239472 (PMC7505428; doi:10.1371/journal.pone.0239472)
Supplement: S1 Appendix — (DOCX) [file pone.0239472.s001.docx]

**Appendix I : Interview questions**

**Potential to Respond**

- What are the events for which the system has a prepared response in the emergency department? **(Event list)**
- How were these events selected? (tradition, regulator requirement, design basis, experience, expertise, risk assessment, industry standard, etc.)? **(Background)**
- Who is responsible for maintaining and evaluating the list? **(Relevance)**
- In everyday clinical work, did you ever feel that the workload exceeded the capacity of the ED? When or in what period of time did you feel this? **(Threshold)**
- When the demand exceeded the ED’s capacity, which situation(s) triggered the need for additional support or external resources? How did you or your ED team members respond to such situation(s)? **(Response list)**
- When the demand exceeds the ED’s capacity in terms of daily surge, how does the entire ED respond to the situation? How fast can the required resources be deployed to support emergency care? **(Speed)**
- Do you have well-prepared emergency response plans or temporary adaptive procedures to deal with the varied situations of demand exceeding capacity? What is the effectiveness of these plans or procedures? **(Response capability)**
- Do you think the prepared surge capacity can sustain a routine daily surge till the ED’s normal operations are resumed? **(Stop rule)**
- How long can the ED surge capacity be sustained to cope with a daily surge? **(Duration)**
- Have you prepared the necessary resources and manpower for routinely verifying or maintaining your emergency response plan and procedures? **(Verification)**

**Potential to Monitor**

- How have the indicators been defined to monitor the changing workload in routine work? **(Indicator list)**
- What is the basis for setting up and revising the monitoring index? **(Relevance)**
- Is monitoring index a single/multiple qualitative/quantitative indicator? **(Indicator characteristic)**
- How often are the indicators updated for monitoring daily operations? **(Measurement frequency)**
- Is there a regular audit or review process for the collection and use of the indicators? **(Organization support)**
- How many indicators do you analyze for an intended purpose? **(Analysis)**
- How do you validate the effectiveness of the indicators? **(Validity)**

**Potential to Anticipate**

- Do you think the unit or person assigned to make future projections has sufficient resources to do the job accurately? **(Expertise)**
- Do you have an existing procedure(s) to anticipate the future threat to the ED? **(Expertise)**
- How and how often do you anticipate future threats? For example, the workload associated with a patient surge exceeding the ED’s capacity. **(Frequency)**
- How are the expectations about future events communicated between staff within the same unit and between different units? **(Communication)**
- Does the system have a clearly formulated model for the future? **(Strategy)**
- Does the anticipation model of ED security have a particular algorithm for predicting the scope and depth of some incidents? **(Model)**
- Is risk awareness part of the organizational culture? **(Culture)**

**Potential to Learn**

- How are the events selected for learning purposes? **(Selection criteria)**

❑Individual departments select independently (no rules)

❑Hospital accreditation standards

❑Events that occur most frequently *or* are the most serious

❑Events that occur most frequently *and* are the most serious

- How much attention does the hospital pay to employees’ suggestion(s)? **(Learning basis)**

❑No response to the feasible suggestion(s)

❑Discuss as soon as possible for all suggestions

❑Respond to the feasible suggestion(s)

❑Respond to all suggestions and require a clearer explanation from the employees

- Does the system try to learn from success as well as from failures? **(Learning basis)**

❑Only from serious failures

❑Only from moderate to mild failure

❑All recent failures

❑Failures and successes

- How do you collect the events for learning? **(Classification)**

❑None

❑Based on department needs

❑Based on hospital needs

❑Based on hospital accreditation needs

- Are there any formal procedures for data collection, analysis, and learning? **(Formalization)**

❑Analysis only without training

❑Possess data analysis and learning but no formal procedures for analysis

❑Formal procedures for analysis and learning in the hospital

❑Additional courses to improve the analysis ability of employees

- How do you learn from and pass on the experiences of handling of general emergency situations? **(Training)**

❑A few people are trained

❑A related group is trained

❑The lessons are embedded into a formal training course and hospital announcement (e.g., official documents)

❑ The lessons are discussed in routine meetings

- Is learning a continuous or discrete (event-driven) activity and for what target group? **(Learning style)**

❑Discrete learning for individual staff members working on the front line only

❑Discrete learning for the working team

❑Discrete learning for all related staff across levels

❑Continuous learning for all staff

- How do you maintain the hospital’s attention on events reporting, analysis, and learning? **(Resource)**

❑Routine evaluation

❑Provision of the reward or incentive program

❑Providing additional relevant information and external visits and training

❑Allocation of sufficient departmental budget

- How many channels or resources for employees to respond/suggestion from the hospital? **(Resource)**

❑None

❑One

❑Two

❑Three or more

- On which level does the learning ability builds up? **(Learning target)**

❑None

❑Individual

❑Collective

❑Organization

- How are lessons learned or implemented? **(Implementation)**

❑Unknown

❑Organizing competition within the hospital

❑Participating competition outside of the hospital

❑Continuous monitoring and routine or non-routine evaluation (procedures)
